# Assessing rodents as carriers of pathogenic *Leptospira* species in the U.S. Virgin Islands and their risk to animal and public health

**DOI:** 10.1038/s41598-022-04846-3

**Published:** 2022-01-21

**Authors:** Camila Hamond, A. Springer Browne, Leah H. de Wilde, Richard L. Hornsby, Karen LeCount, Tammy Anderson, Tod Stuber, Hannah M. Cranford, Stephanie K. Browne, Gerard Blanchard, David Horner, Marissa L. Taylor, Michael Evans, Nicole F. Angeli, Joseph Roth, Kristine M. Bisgard, Johanna S. Salzer, Ilana J. Schafer, Brett R. Ellis, David P. Alt, Linda Schlater, Jarlath E. Nally, Esther M. Ellis

**Affiliations:** 1 APHIS, U.S. Department of Agriculture, National Veterinary Services Laboratories, Ames, IA USA; 2 U.S. Department of Agriculture, NCAH Leptospira Working Group, Ames, IA USA; 3 Epidemic Intelligence Service, Division of Scientific Education and Professional Development, Centers for Disease Control and Prevention, Atlanta, GA USA; 4 U.S. Virgin Islands Department of Health, Christiansted, VI USA; 5 Domestic Animal Health Analytics Team, Center for Epidemiology and Animal Health, United States Department of Agriculture, Fort Collins, CO USA; 6 Infectious Bacterial Diseases Research Unit, National Animal Disease Center-USDA-ARS, 1920 Dayton Avenue, Ames, IA 50010 USA; 7 Council for State and Territorial Epidemiologists, Atlanta, GA USA; 8 U.S. Department of Agriculture, Wildlife Services, Charlotte Amalie, VI USA; 9 National Park Service, St. John, VI USA; 10 U.S. Fish and Wildlife Service, Fredericksted, VI USA; 11 U.S. Virgin Islands Department of Planning and Natural Resources, Fredericksted, VI USA; 12 Division of Scientific Education and Professional Development, Centers for Disease Control and Prevention, Atlanta, GA USA; 13 Bacterial Special Pathogens Branch, Division of High-Consequence Pathogens and Pathology, Centers for Disease Control and Prevention, Atlanta, GA USA

**Keywords:** Microbiology, Diseases

## Abstract

Leptospirosis is a global zoonotic disease caused by pathogenic bacteria of the genus *Leptospira*. We sought to determine if rodents in U.S. Virgin Islands (USVI) are carriers of *Leptospira*. In total, 140 rodents were sampled, including 112 *Mus musculus* and 28 *Rattus rattus*. A positive carrier status was identified for 64/140 (45.7%); 49 (35.0%) were positive by dark-field microscopy, 60 (42.9%) by culture, 63 (45.0%) by fluorescent antibody testing, and 61 (43.6%) by real-time polymerase chain reaction (rtPCR). Molecular typing indicated that 48 isolates were *L*. *borgpetersenii* and 3 were *L*. *kirschneri*; the remaining nine comprised mixed species. In the single culture-negative sample that was rtPCR positive, genotyping directly from the kidney identified *L. interrogans*. Serotyping of *L*. *borgpetersenii* isolates identified serogroup Ballum and *L. kirschneri* isolates as serogroup Icterohaemorrhagiae. These results demonstrate that rodents are significant *Leptospira* carriers and adds to understanding the ecoepidemiology of leptospirosis in USVI.

## Introduction

Leptospirosis is a zoonosis of global distribution caused by pathogenic species in the genus *Leptospira* that infects people, wildlife, and domestic animals^1^. Rodent species act as reservoir hosts without clinical disease from *Leptospira,* which colonize renal tubules and are excreted through urine contaminating water and soil where it can survive for weeks^2^. Humans are incidental hosts and exposure occurs by direct contact with infected animals or indirectly through contact with contaminated water or soil. Human leptospirosis ranges in severity from a mild, self-limited febrile illness to a fulminant life-threatening illness^3^. Leptospirosis in domestic animals is characterized by similar acute clinical features, but persistent chronic infection in animals can occur^4,5^.

Acute infections are more common in low-resource, tropical and subtropical locations where outbreaks can occur after natural disasters, such as hurricanes and concurrent rainfall and flooding^1^. The U.S. Virgin Islands (USVI) is a U.S. territory located in the Caribbean Ocean with three main islands, St. Croix (STX), St. Thomas (STT), and St. John (STJ), totaling ~ 133 square miles^6^, with a population of 106,405 persons^7^. The climate is tropical, with average high temperature ranges of 82°F to 90°F, and average rainfall of 43 inches per year. USVI was directly struck by Category 5 Hurricanes Irma and Maria in September 2017, generating record-breaking rainfalls and flooding. Afterwards, the Virgin Islands Department of Health documented the first-known human cases of leptospirosis on the islands^6^. A follow-up seroprevalence study among residents reported evidence of exposure to *Leptospira*, with highest reacting titers to serogroups Icterohaemorrhagiae, Australis, Canicola, Pyrogenes, Tarassovi, Autumnalis, Bataviae, Djasiman, Ballum and Sejröe (Esther Ellis, USVI Department of Health, Personal Communication 2019). Assessment of animal leptospirosis in USVI has been limited to a single serologic study of small ruminants conducted in 1992, which showed reactivity to serogroups Autumnalis, Ballum, Bataviae, Australis, Canicola, Icterohaemorrhagiae, Sejröe and Pyrogenes^8^.

Rodents are a principal reservoir host for the transmission of leptospirosis^9,10^. Given our limited understanding of leptospirosis disease transmission in USVI, this investigation sought to determine the extent to which wild rodents act as carriers of leptospires and identify any associated species of *Leptospira*. Such information is critical for efficacious surveillance, control, and prevention strategies.

## Materials and methods

### Sample collection

**Figure 1:**
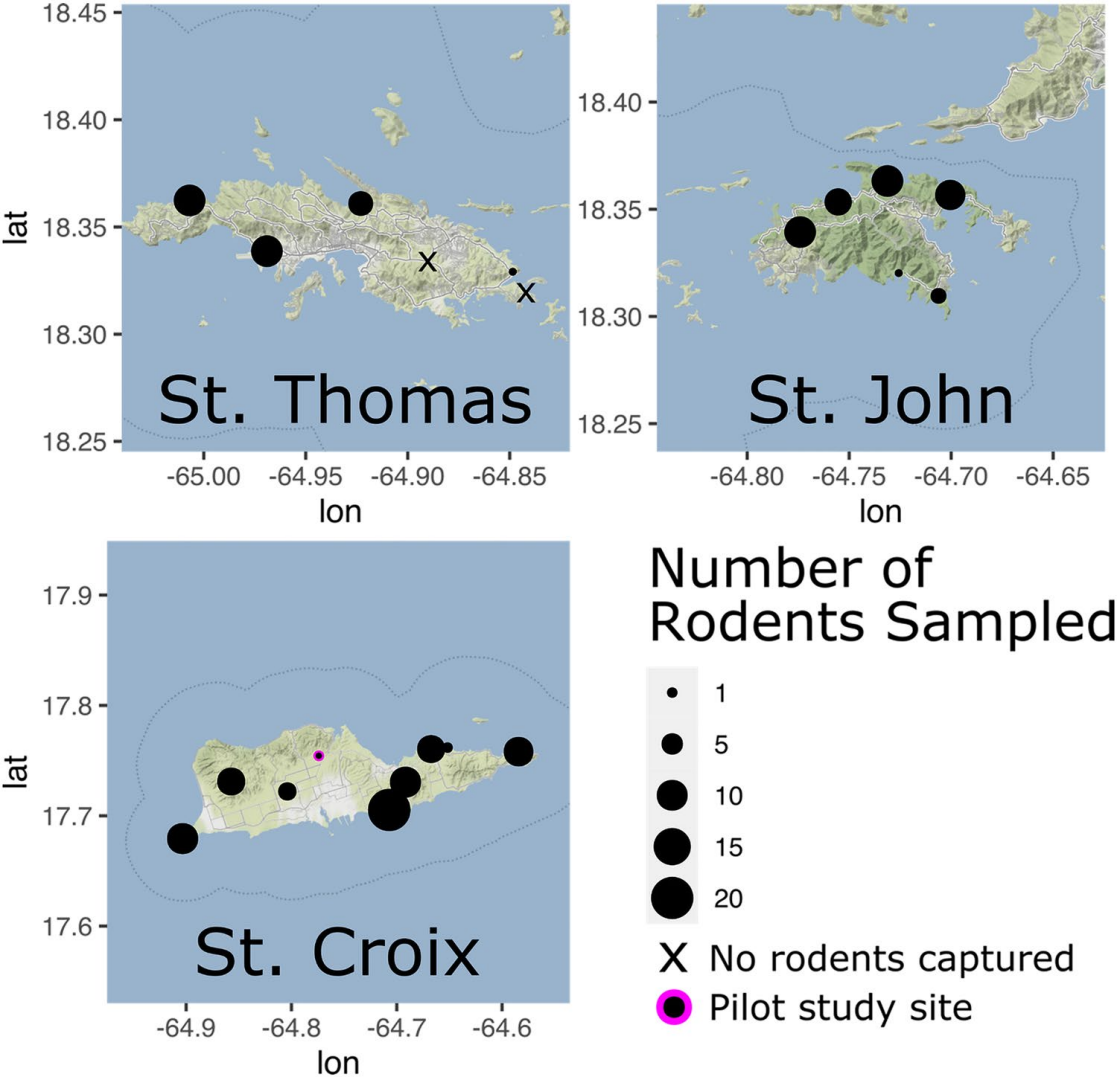
Map of U.S. Virgin Islands showing the sampled areas among the three islands: St. Croix, St. Thomas, and St. John. Black dots identify each location, with the size correlating to the numbers of rodents sampled. Map base layer available under CC BY 3.0 license: http://maps.stamen.com/terrain/#10/18.0114/-64.7823.

Field activities and euthanasia procedures were in accordance with CDC IACUC Protocol Numbers 2879SALMULX-A4 and AVMA Guidelines for Euthanasia of Animals^11^, and in compliance with the ARRIVE guidelines. A pilot study was performed in STX at a single unique study site in September 2019 to assess logistics of field sampling and laboratory processing and shipment. In total, 39 Sherman Traps® (H.B. Sherman Traps, Tallahassee, Florida, USA) were deployed, and three *Mus musculus* were sampled. The cross-sectional field study was carried out during June 15–June 30, 2020 and employed single sample events at 20 different study sites from three islands as follows: eight in STX, six in STT and six in STJ (Table 1 and Fig. 1). Trapping consisted of ten 6 × 6 × 18-inch (15 × 15 × 46 cm) Tomahawk® live traps (Tomahawk Live Trap Co, Tomahawk, Wisconsin, USA) and 80 Sherman Traps®, 15 m apart at each study site. For bait, oat and peanut butter mixture were used in the Sherman Trap®; Vienna sausage was used in Tomahawk traps. Traps were placed in rural areas (e.g., farms, parks, or bush) throughout USVI in the evening and collected at dawn for a single sampling event per site.

**Table 1:** Number of rodents captured and sampled, by island and field site as shown in Fig. 1.

Island	Field site	Rodents captured			Rodents sampled*		
		*Mus muscularis* captured	*Rattus rattus* captured	Total captured	*Mus muscularis* sampled	*Rattus rattus* sampled	Total sampled
St. Croix (STX)	Concordia*	3	0	3	3	0	3
	Jolly Hill	8	0	8	8	0	8
	Sandy Point	8	0	8	8	0	8
	Haypenny Beach^†^	31	2	33	18	2	20
	Lower Love	4	0	4	4	0	4
	Cramer Park	9	0	9	9	0	9
	Prune Bay	1	2	3	1	2	3
	Recovery Hill	13	3	16	7	3	10
	Southgate	7	1	8	7	1	8
St. Thomas (STT)	Stumpy Bay	13	8	21	5	5	10
	Magens Bay	6	0	6	6	0	6
	STT Airport	31	0	31	10	0	10
	Tutu	0	0	0	0	0	0
	Vessup Beach	0	0	0	0	0	0
	Red Hook Point	2	0	2	2	0	2
St. John (STJ)	Gifft Hill Landfill	20	6	26	4	6	10
	Cinnamon Bay	7	0	7	7	0	7
	Lameshur Bay	0	1	1	0	1	1
	Salt Pond	2	0	2	2	0	2
	Brown Bay	6	3	9	6	3	9
	Annaberg Plantation	13	5	18	5	5	10
Totals		184	31	215	112	28	140

*Pilot study site sampled on September 3, 2019; in total, 39 Sherman traps deployed.

^†^Because of laboratory resources and field safety, sampling was limited to 10 rodents per site after sampling at Haypenny Beach.

Because of finite laboratory resources and field and animal safety in a tropical climate, sampling was limited to a maximum of 10 rodents per field site after capture of 33 rodents in Haypenny Beach during the second day of the study (Table 1). Thereafter, any rodents captured surplus to sampling needs were immediately released back into their environment. To avoid selection bias, if more than 10 rodents were captured at a sampling site, traps with captured rodents were randomly selected using a random number generator for sampling. Species was confirmed visually, and selection preferred a 50/50 split between *Mus musculus* and *Rattus rattus*, if possible.

Rodents were rapidly anesthetized with isoflurane until euthanasia by cervical dislocation. Sex, mass, and morphometrics (e.g., total body length, ear, hind foot, and tail lengths) were recorded. Blood samples were collected by cardiac puncture and stored on ice. Samples were centrifuged (15,000×*g* for 15 min) and serum collected and stored at −20 °C. Frozen serum was then transported to the National Veterinary Services Laboratories, APHIS, U.S. Department of Agriculture (USDA), Ames, Iowa. One kidney from each rodent was removed using aseptic technique by necropsy and immediately stored in Hornsby-Alt-Nally (HAN) media^12^, then transported by overnight delivery services at ambient temperature to the National Animal Disease Center, ARS, USDA, Ames, Iowa.

### Microscopic agglutination test (MAT)

The microscopic agglutination test (MAT) was performed according to World Organization for Animal Health guidelines using a panel of 18 antigens representative of 15 serogroups (Supplementary Table 1)^13^. A titer was considered positive at ≥ 1:100.

### Kidney sample processing

The kidney was macerated in 9 mL of HAN media in 710 mL Whirl–Pak® bags (Nasco).

### Dark-field microscopy (DFM)

Ten microliters of the macerate were placed on a microscope slide and cover-slipped. Ten fields were examined by DFM (× 200 and × 400) for leptospires.

### Culture

One mL of the kidney macerate was used to inoculate 9 mL HAN liquid medium and 200 µl of this dilution was inoculated into 5 mL of three different media: semisolid T80/40/LH^14^ that was incubated at 29℃, and 5 mL of liquid and semisolid HAN, which were incubated at 37℃ in 3% CO_2_^12^. Semisolid cultures were observed using a lighted black background to examine for development of a Dinger’s zone (DZ), and if noted, were confirmed as positive by DFM, at days 3 and 5, weekly for one month, and monthly thereafter for six months. From the positive cultures, average time for a DZ to appear was noted. Inoculated tubes of liquid HAN were examined daily by DFM for leptospires from day 3 to day 8.

### Fluorescent Antibody Testing (FAT)

A 10 µl aliquot of the kidney macerate was placed on a glass slide within a 7 mm well, in duplicate, and FAT performed as previously described^15^.

### DNA extraction

DNA was extracted from 500 μL of kidney macerate using the Maxwell RSC Purefood Purification Pathogen kit (Promega Corporation, Madison, Wisconsin, USA), following manufacturer's instructions, but using 1 h of incubation with lysis buffer A and a 100μL elution volume. For cultures, DNA was extracted from 5 mL of each isolate in HAN media, which was harvested by centrifugation at 10,000×*g* for 15 min.

### Real-time polymerase chain reaction (rtPCR)

After DNA extraction from 500 µl of kidney macerate, 5 µl was used for rtPCR. The *lipL32* gene was amplified using a set of primers and protocol as described previously: LipL32-47Fd (5′-GCATTACMGCTTGTGGTG -3′) and LipL32-301Rd (5′-CCGATTTCGCCWGTTGG -3′), the probe LipL32-189P (6-carboxyfluorescein [FAM]-5′-AA AGC CAG GAC AAG CGC CG-3′-black hole quencher 1 [BHQ1]), using PerfeCTa qPCR ToughMix®, Low ROX™ (Quanta Biosciences, Gaithersburg, Maryland, USA)^16,17^. To control for rtPCR inhibitors, the TaqMan® (Thermo Fischer Scientific, Waltham, MA, USA) Exogenous Internal Positive Control (IPC) was added to the master mix to confirm DNA amplification and detect false negatives and to qualitatively detect presence of amplification inhibitory substances in a sample. If IPC negative samples indicated the sample contained rtPCR inhibitors, samples were diluted 1:10 and rtPCR repeated. If the diluted sample was still negative for IPC, a new DNA extraction was performed. All samples were assayed in triplicate and considered positive when duplicate or triplicates were positive with *Ct* value < 40^16,17^.

### Molecular and serological typing of cultured *Leptospira* species

Concentration of reconstituted genomic DNA was determined by Qubit® (Qubit dsDNA BR assay, Qubit 3.0 fluorometer, Invitrogen, Carlsbad, CA, USA). Whole-genome sequence of all cultures was obtained (MiSeq Desktop Sequencer, 2 × 250 v2 paired-end chemistry and the Nextera XT DNA Library Preparation Kit, Ilumina, San Diego, California) per manufacturer’s instructions and draft assemblies of each genome were mined to retrieve full length *secY* coding regions which were analyzed with Geneious Prime 2020.2.2 (geneious.com). Consensus sequences of 522 bp were then compared with sequences in GenBank using BLAST (Basic Local Alignment Search Tool). *secY* sequences for USVI rodent isolates were deposited in the National Center for Biotechnology Information (NCBI) database, accession numbers MZ241244–MZ241294. A phylogenetic tree was made with Geneious Prime 2020.2.2 using the neighbor‐joining method, with the Tamura‐Nei nucleotide substitution model (https://www.geneious.com/).

Isolates were serotyped by MAT using a panel of polyclonal rabbit reference antisera representing 13 serogroups; Australis, Autumnalis, Ballum, Bataviae, Canicola, Grippotyphosa, Hebdomadis, Icterohaemorrhagiae, Mini, Pomona, Pyrogenes, Sejröe, and Tarassovi (National Veterinary Services Laboratories, APHIS, USDA, Ames, Iowa) (Supplementary Table 2). The serogroup for each isolate was assigned according to the antiserum that gave the highest agglutination titer^18^.

### Genotyping of *Leptospira* directly from kidney samples

The *secY* housekeeping gene was amplified with the primers *secY*_F (5′ -ATGCCGATCATTTTTGCTTC-3′) and *secY*_R (5′-CCGTCCCTTAATTTTAGACTTCTTC-3′)^19^. PCR products were then purified and labeled using the Big Dye Terminator v3.1 cycle sequencing reagent (Applied Biosystems, Foster City, California, USA). Sequencing was performed using the ABI 3130XL Genetic Analyzer. Sequence data were analyzed with DNAStar’s Lasergene sequence analysis software. Consensus sequences were compared with available sequences in the GenBank database using BLAST. Phylogenetic analyses were performed as described previously. The *secY* sequence was deposited in NCBI, accession number MZ241295.

### Evaluation of virulence

All animal experimentation was conducted in accordance with protocols as reviewed and approved by the Animal Care & Use Committee at the National Animal Disease Center (ARS-2018-745), and as approved by USDA institutional guidelines. Five representative rodent isolates of *L*. *borgpetersenii* (designated LR45, LR47, LR59, LR88, and LR131) were propagated in liquid HAN medium^12^ supplemented with 0.4% rabbit serum at 37 °C in 3% CO_2_ and evaluated for virulence by intraperitoneal injection into five groups of four golden Syrian hamsters (*Mesocricetus auratus*), as previously described^15^. One group of four animals was also inoculated through the conjunctival route with 10^8^ of strain LR131 in 10 µl of HAN medium, which was applied to the conjunctival membrane of the left eye, as previously described^20^. Liver and kidney tissue were harvested for culture, FAT, and lipL32 rtPCR when hamsters met euthanasia criteria attributable to clinical signs of infection including weight loss, lethargy, bloody discharge from the nose or urogenital tract, and sudden death^21^.

### Statistical analyses

Comparison of culture results in HAN and T80/40/LH media were assessed using the Students T-test. Analysis was conducted using SPSS statistical software (SPSS Inc., Chicago, Illinois, USA), and results were considered significant when *p* < 0.05.

## Results

### Rodent survey

In total, 140 rodents were sampled, including *Mus musculus* (n = 112) and *Rattus rattus* (n = 28): 73 in STX, 28 in STT and 39 in STJ (Table 1, Fig. 1). Overall trap success was 11.7% (215 rodents captured for 1,839 trap-nights deployed); rodents were sampled at 19/21 study sites (i.e., one pilot study site; 20 cross-sectional survey sites), and no rodents were captured at two study sites in St. Thomas. More female rodents (n = 82) were sampled than males (n = 58). Among sexually mature females, 3/80 (4%) were pregnant.

### Seroprevalence

Of 112/140 (80.0%) sera tested by MAT, 60/112 (53.6%) were positive (titer ≥ 1:100). Twenty-eight (20.0%) sera samples were of inadequate volume to perform MAT. Of 60 positive sera samples, 51 had sufficient volumes remaining to determine titers against reacting serogroups (Table 2). The most frequent highest-reacting titer was to serogroup Ballum (30/51, 58.8%), followed by Icterohaemorrhagiae (5/51, 9.8%), Hebdomadis (5/51, 9.8%), Djasiman (2/51, 3.9%), Cynopteri (2/51, 3.9%) and Australis (1/51, 2.0%) (Table 2). Equivalent high titers were observed to more than one serogroup in six samples; three reacted with both Australis and Hebdomadis (LR119 with a titer of 1:100, LR138 and LR140 with a titer of 1:400), one (LR34) reacted to both Sejröe and Ballum (titer of 1:1600), one (LR71) reacted to both Autumnalis and Icterohaemorrhagiae (titer of 1:100) and one (LR113) with both Hebdomadis and Cynopteri (titer of 1:100).

**Table 2:** Titers of rodent samples reactive with serogroups of Leptospira*.

Titer Serogroup	100	200	400	800	1600	3200	6400	12,800	Total
Australis	–	1	–	–	–	–	–	–	1
Ballum	3	3	3	2	10	7	2	–	30
Cynopteri	1	1	–	–	–	–	–	–	2
Djasiman	1	1	–	–	–	–	–	–	2
Hebdomadis	1	2	–	–	2	–	–	–	5
Icterohaemorrhagiae	–	–	–	1	–	2	–	2	5
Total	6	8	3	3	12	9	2	2	45

*Not included are those six samples most reactive to two different serogroups and as reported in Supplementary Table 3.

### Detection of leptospires in rodent kidney

**Figure 2:**
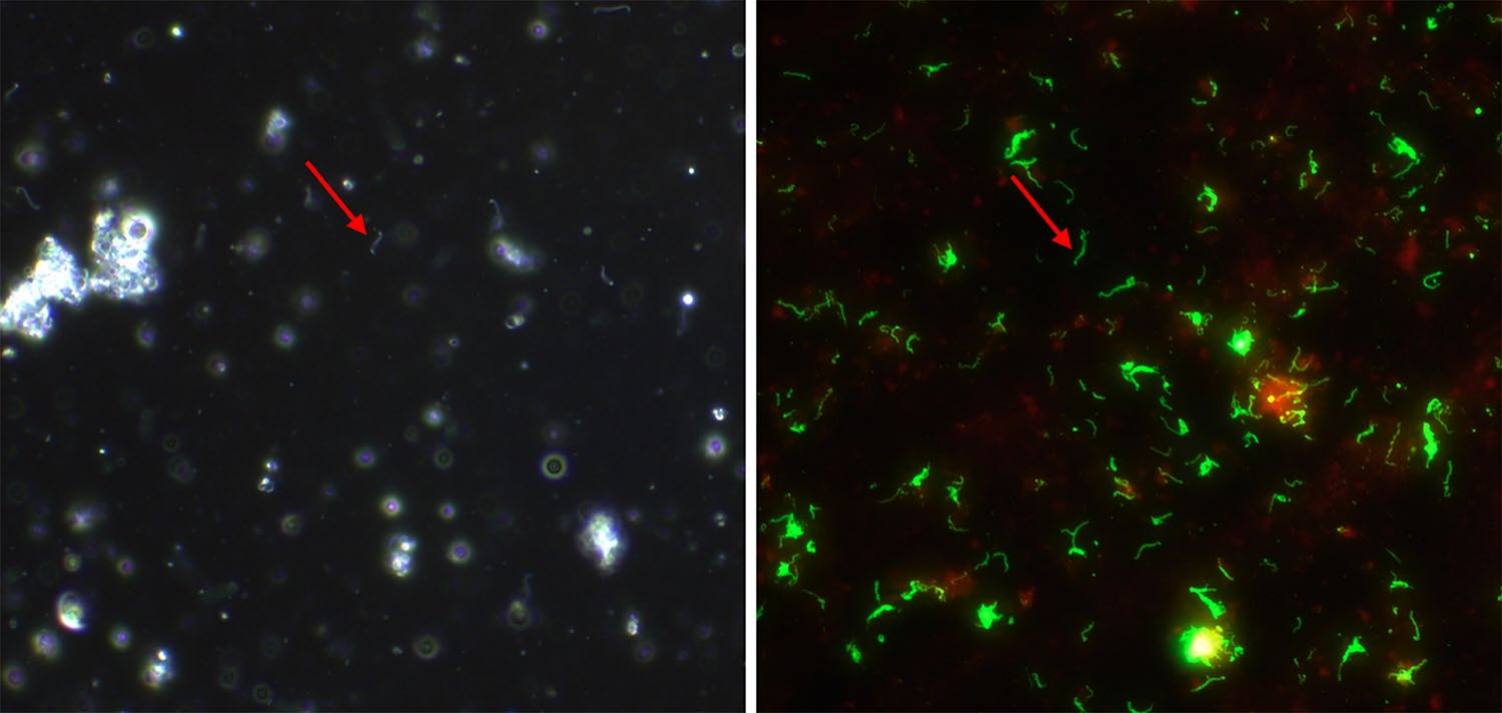
Representative images of a rodent kidney sample that was positive for leptospires by (**A**) dark-field microscopy and (**B**) fluorescent antibody test (FAT). Red arrows indicate leptospires. Original magnification 400×.

Forty-nine (35.0%; 49/140) kidney samples were positive for *Leptospira* by DFM (Table 3): 32/73 (43.8%) in STX, 5/28 (17.9%) in STT and 12/39 (30.8%) in STJ. By FAT, 63/140 (45.0%) were positive (Table 3): 39/73 (53.4%), 6/28 (21.4%) and 18/39 (46.2%) on STX, STT and STJ respectively. All samples positive by DFM were positive by FAT (Fig. 2). rtPCR for *lipL32* detected 61 (43.6%) positive kidneys (Table 3): 38/73 (52.1%) in STX, 6/28 (21.4%) in STT and 17/39 (43.6%) in STJ. The average *Ct* value of positive samples was 24.8 ± 4.1 (95% CI).

**Table 3:** Detection of *Leptospira* in different rodent species from different study sites by dark-field microscopy (DFM), fluorescent antibody test (FAT), real-time polymerase chain reaction (rtPCR), and culture.

Species	DFM	FAT	rtPCR	Culture
*Mus musculus*	47/112 (41.9%)	56/112 (50%)	52/112 (46.4%)	51/112 (45.5%)
*Rattus rattus*	2/28 (7.1%)	7/28 (25%)	9/28 (32.1%)	9/28 (32.1%)
Total	49/140 (35%)	63/140 (45%)	61/140 (43.7%)	60/140 (42.8%)

*Leptospira* species were isolated by culture from 60 (42.9%) individual kidneys (Table 3): 37/73 (50.7%) in STX, 6/28 (21.4%) in STT and 17/39 (43.6%) in STJ. Fifty-seven isolates were recovered in all three media including liquid and semisolid HAN at 37℃ and T80/40/LH at 29℃ and three isolates were recovered in only liquid and semisolid HAN media. For positive cultures, the average time for a DZ to appear in semisolid HAN medium was 7.4 ± 3.3 (95% CI) days; whereas, the average number of days required for T80/40/LH medium was 17.2 ± 5.1 (95% CI). HAN media demonstrated a significant difference (*p* < 0.001) in the fewer days required from primary inoculation to development of a visible DZ. Positive cultures were detected in HAN liquid media at 5 ± 1 days postinoculation, as defined by detection of a single leptospire by dark-field microscopy.

A positive carrier status was identified for leptospires in 64 (45.7%) rodents as defined by a positive result in any of the assays used; 57 (89.1%) were positive by culture, FAT and rtPCR; three samples were positive by culture and rtPCR; three samples were positive only by FAT and one sample was positive only by rtPCR. Notably, two rodents identified as carriers were seronegative. All data is presented in Supplementary Table 3.

### Molecular and serotyping of *Leptospira* isolates

**Figure 3:**
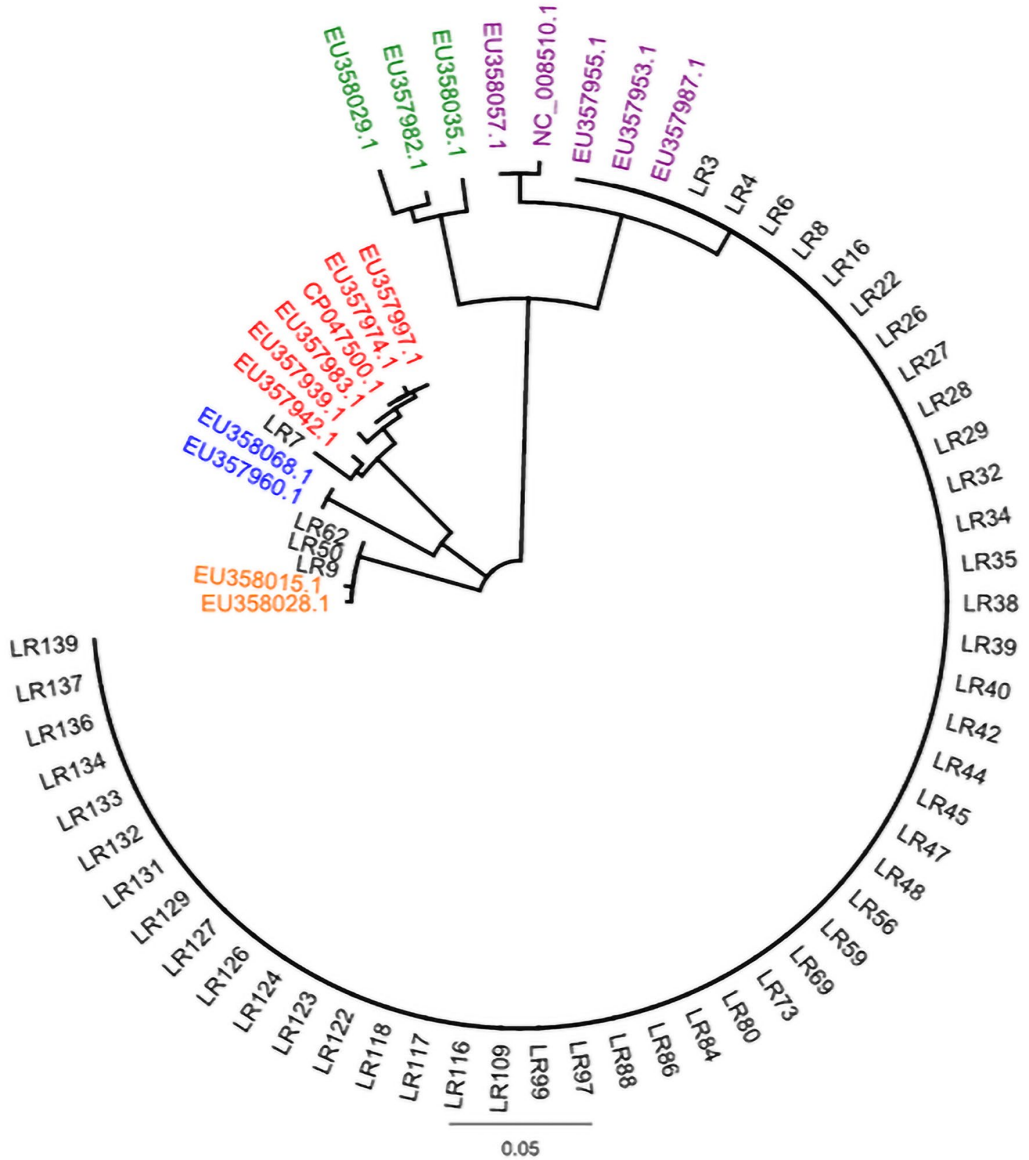
Phylogeny of *Leptospira* isolates based on *secY* gene sequence (522 bp) analysis using the neighbor-joining method. U.S. Virgin Islands isolates of *Leptospira* from rodents are annotated as LR and colored black, whereas accession numbers are provided for reference strains of *L. borgpetersenii* from different hosts (purple), *L. santarosaii* (green), *L. kirschneri* (orange), and *L. noguchii* (blue). Note sample LR7 which was genotyped directly from kidney and clades with reference strains of *L. interrogans* (red).

Molecular typing indicated that 48/60 isolates showed 100% *secY* identity with *L. borgpetersenii* and clustered with *L. borgpetersenii* serovar Polonica (EU357987.1), *L. borgpetersenii* serovar Ballum (EU357953.1) and *L. borgpetersenii* serovar Castellonis (EU357955.1); three additional isolates showed 99.8% identity with *L. kirschneri* (Fig. 3). The remaining 9/60 isolates were not readily identified to species level, and likely contain a mixed population of species of both *L*. *borgpetersenii* and *L*. *kirschneri* (data not shown). Serotyping of 48 isolates of *L*. *borgpetersenii* indicated that all belong to serogroup Ballum, and three isolates of *L. kirschneri* belong to serogroup Icterohaemorrhagiae. All rodent isolates obtained from STT and STJ were classified as *L. borgpetersenii* serogroup Ballum. However, cultures of *Leptospira* from rodent isolates in STX included those classified as *L. borgpetersenii* serogroup Ballum and *L. kirschneri* to serogroup Icterohaemorrhagiae, as well as cultures that were potentially mixed species.

### Genotyping of *Leptospira* directly from kidney

One kidney sample designated LR7 was positive only by rtPCR. Subsequent PCR amplification of partial sequence of *secY* followed by sequencing and BLAST/NCBI comparisons with GenBank indicated 100% identity with *L. interrogans* (Fig. 3).

### Evaluation of virulence

Intraperitoneal inoculation of all hamsters with 10^8^ leptospires of each of five strains resulted in acute disease requiring all to be euthanized at 5 days post-infection. Liver and kidney samples from each group were positive by culture, FAT and *lipL32* rtPCR. A single group inoculated by the conjunctival route using isolate LR131 showed acute disease by 11 days post-infection; liver and kidney samples from this group also tested positive by culture, FAT and *lipL32* rtPCR.

## Discussion

Sampling of USVI rodents determined that 45.7% (64/140) were carriers of pathogenic *Leptospira* species (*L. borgpetersenii*, *L. kirschneri*, or *L. interrogans*) as defined by at least one positive FAT, rtPCR, or culture.

Seroprevalence for rodents in this study was relatively high (53.6%; 60/112), compared with previous findings from Central American countries including Barbados (32.6%)^22^, Grenada (24.5%)^23^, Guadeloupe (32%)^22^, and Trinidad (20.5%)^24^. Reactivity to serogroup Ballum was most frequently detected and differs from other studies with rodents in the same region, which reported reactivity primarily to serogroup Icterohaemorrhagiae^9,22–24^. Previous work demonstrated that goats in USVI were also reactive to serogroups Ballum and Icterohaemorrhagiae^8^. However, a positive serology in reservoir hosts of infection is indicative only of exposure and not active disease^4^.

Culture is the definitive diagnostic assay to detect shedding of leptospires, though it can have low sensitivity due to the fastidious nature of *Leptospira*^4^. The use of the newly described HAN media allowed for recovery of *Leptospira* species in a significantly shorter time frame at 37℃, compared with T80/40/LH at 29℃, and likely attributable to growth factors and conditions that more closely emulate the in vivo environment to support metabolic requirements of in vivo derived leptospires^12^. Recovery of isolates from rodent hosts allows for their more complete characterization at the genotypic and phenotypic level, and their use for enhanced diagnostic (i.e., animal and human) or bacterin-based vaccination strategies of animals to limit zoonotic transmission.

We attributed 80% of active rodent infections to *L. borgpetersenii* serogroup Ballum and 5% to *L. kirschneri* serogroup Icterohaemorrhagiae. One *lipL32* rtPCR positive, but culture negative sample was genotyped as *L. interrogans* directly from kidney^25^. Among nine culture positive samples, species identification was not readily apparent and likely attributable to mixed populations of species. Further analysis of these mixed bacterial samples is underway to obtain clonal isolates, along with a comprehensive analysis of genomes of all recovered isolates. Notably, all mixed and *L*. *kirschneri* carriage in rodents were limited to those rodents trapped on St. Croix island. St. Croix rodent populations may have developed a unique carrier population of *Leptospira* because of its remote location and agricultural landscape.

Identification of *L. borgpetersenii*, *L. kirschneri*, and *L. interrogans* in kidneys of rodents in USVI is similar to recent findings in mongoose from USVI which were carriers of *L. borgpetersenii*, *L*. *kirschneri* and *L. interrogans* species^26^. However, and in contrast to USVI mongoose which were carriers of *L*. *borgpetersenii* serogroup Sejroe, USVI rodents were carriers of *L*. *borgpetersenii* serogroup Ballum. Seroprevalence investigations have also implicated serogroups Ballum in human leptospirosis in USVI (Esther Ellis, USVI Department of Health, personal communication, 2019) and livestock leptospirosis in USVI^27^ and across the Caribbean, including Puerto Rico^28^, Barbados^29^, Cuba^30^, Martinique^25^, Guadeloupe^25,31^ and Jamaica^32^; serogroup Ballum is also detected in other regions, such as New Caledonia^33^ and Australia^34,35^. Virulence of *L. borgpetersenii* serogroup Ballum from rodents in USVI was confirmed in the hamster model of leptospirosis, which emulates pathology associated with acute lethal forms of human leptospirosis and similar to that observed for serogroup Ballum isolates from Puerto Rico^28^ and New Caledonia^36^. Positive MAT titers against serogroup Ballum are indicative of human exposure, although its role in human disease has yet to be determined^28^. Rodents from rural and urban areas of Puerto Rico are carriers of *L. interrogans* and *L. borgpetersenii*^28,37^; rodents from Martinique and Guadeloupe are carriers of *L. borgpetersenii*, *L. interrogans,* and *L. kirschneri*, and rodents from urban areas of New Orleans are carriers of *L. borgpetersenii*, *L. interrogans*, and *L. kirschneri*^38^.

Two species of introduced (non-native) rodents were trapped; *Mus musculus* and *Rattus rattus*^39^. The rodent species diversity observed in rural areas in USVI was not as high as that observed in other rural settings in nearby countries^40,41^; a predominance of *Rattus* species are frequently noted within the Caribbean, including Grenada, Guadeloupe, Trinidad and Barbados^9,22–24^, which is in contrast to Puerto Rico^37^ and results reported here for USVI in which trapped rodents were predominantly *M. musculus* (80%).

Exposure to rodents is associated with an increased risk of leptospirosis^42^. Serogroup Ballum, the principal reservoir of which is mice^36^, is increasingly reported in human infections^31,43^ and our results highlight the need for rodent control to limit effects of leptospirosis^36^. Human leptospirosis infections usually reflect serogroups maintained by local animal populations highlighting the need for serovar-specific vaccine development in high-risk populations. Serogroup Ballum is not included in commercial bacterins for animals despite evidence of infection and its association with poor animal reproductive performance^8,44^.

USVI’s climate is typical of maritime tropical environments, with warm and stable temperatures and steady winds. Intense rainfall events generally occur in the form of tropical depressions, storms, or hurricanes. Occurrence of natural disasters and deficiencies in sanitary infrastructure can create a favorable environment for rodent proliferation and increase risk for infection^45^. The first documented case of human leptospirosis was identified in USVI after Hurricanes Irma and Maria, and associated with exposure to flood water and occupation of buildings with evidence of rodent infestation^6^. Research should be conducted in rural and urban USVI areas to identify region-specific risk factors for infection. Leptospirosis is a reemerging disease of public health importance with respect to morbidity and mortality both in humans and animals.

Limitations of this study include using a cross-sectional design that does provide a geographically encompassing assessment of rodents throughout USVI, but in a limited time frame (i.e., two weeks). Rodents can be transient carriers of *Leptospira*, but we did not control for seasonal variation.

In conclusion, this study confirms the presence of three species of pathogenic *Leptospira* (*L. borgpetersenii*, *L. kirschneri*, and *L. interrogans*) among USVI’s rodent populations. Local isolates of *L. borgpetersenii* serogroup Ballum and *L. kirschneri* serogroup Icterohaemorrhagiae should be included in MAT diagnostic panels for human and domestic animal samples from USVI, to increase capacity for disease detection in humans and animals. Control, public health surveillance, and prevention efforts need to be multidisciplinary and multisectoral, making it a prime candidate for the One Health approach.

## Supplementary Information

Supplementary Information 1.

Supplementary Information 2.

Supplementary Information 3.
