# Supplementary material for: Assessing rodents as carriers of pathogenic Leptospira species in the U.S. Virgin Islands and their risk to animal and public health
Source: Sci Rep. 2022 Jan 21;12:1132. doi: 10.1038/s41598-022-04846-3 (PMC8782869; doi:10.1038/s41598-022-04846-3)
Supplement: Supplementary file 1 — Supplementary Information 1. [file 41598_2022_4846_MOESM1_ESM.docx]

**Supplementary Table 1:** Panel of antigens used in the microscopic agglutination test (MAT)

| **Species** | **Serogroup** | **Serovar** | **Strain** |
| --- | --- | --- | --- |
| *L. interrogans* | Australis | Bratislava | Jez Bratislava |
| *L. interrogans* | Australis | Australis | Ballico |
| *L. interrogans* | Autumnalis | Autumnalis | Akiyami A |
| *L. borgpetersenii* | Ballum | Ballum | S 102 |
| *L. interrogans* | Bataviae | Bataviae | Van Tienen |
| *L. interrogans* | Canicola | Canicola | H. Utrecht IV |
| *L. kirschneri* | Cynopteri | Cynopteri | 3522C |
| *L. interrogans* | Djasiman | Djasiman | Djasiman |
| *L. interrogans* | Grippotyphosa | Grippotyphosa | Andaman |
| *L. interrogans* | Hebdomadis | Hebdomadis | Hebdomadis |
| *L. interrogans* | Icterohaemorrhagiae | Copenhageni | M 20 |
| *L. interrogans* | Mini | Szwajizak | Szwajizak |
| *L. interrogans* | Pomona | Pomona | Pomona |
| *L. interrogans* | Pyrogenes | Pyrogenes | Salinem |
| *L. interrogans* | Sejröe | Hardjo | Hardjoprajitno |
| *L. borgpetersenii* | Sejröe | Sejröe | M 84 |
| *L. borgpetersenii* | Tarassovi | Tarassovi | Perepelicin |
| *L. tipperaryensis* | ND | Room 22 | GWTS#1 |

ND: Not determined
